# Supplementary figures and images for: Functional characterization of Cinnamate 4-hydroxylase gene family in soybean (Glycine max)
Source: PLoS One. 2023 May 15;18(5):e0285698. doi: 10.1371/journal.pone.0285698 (PMC10184913; doi:10.1371/journal.pone.0285698)

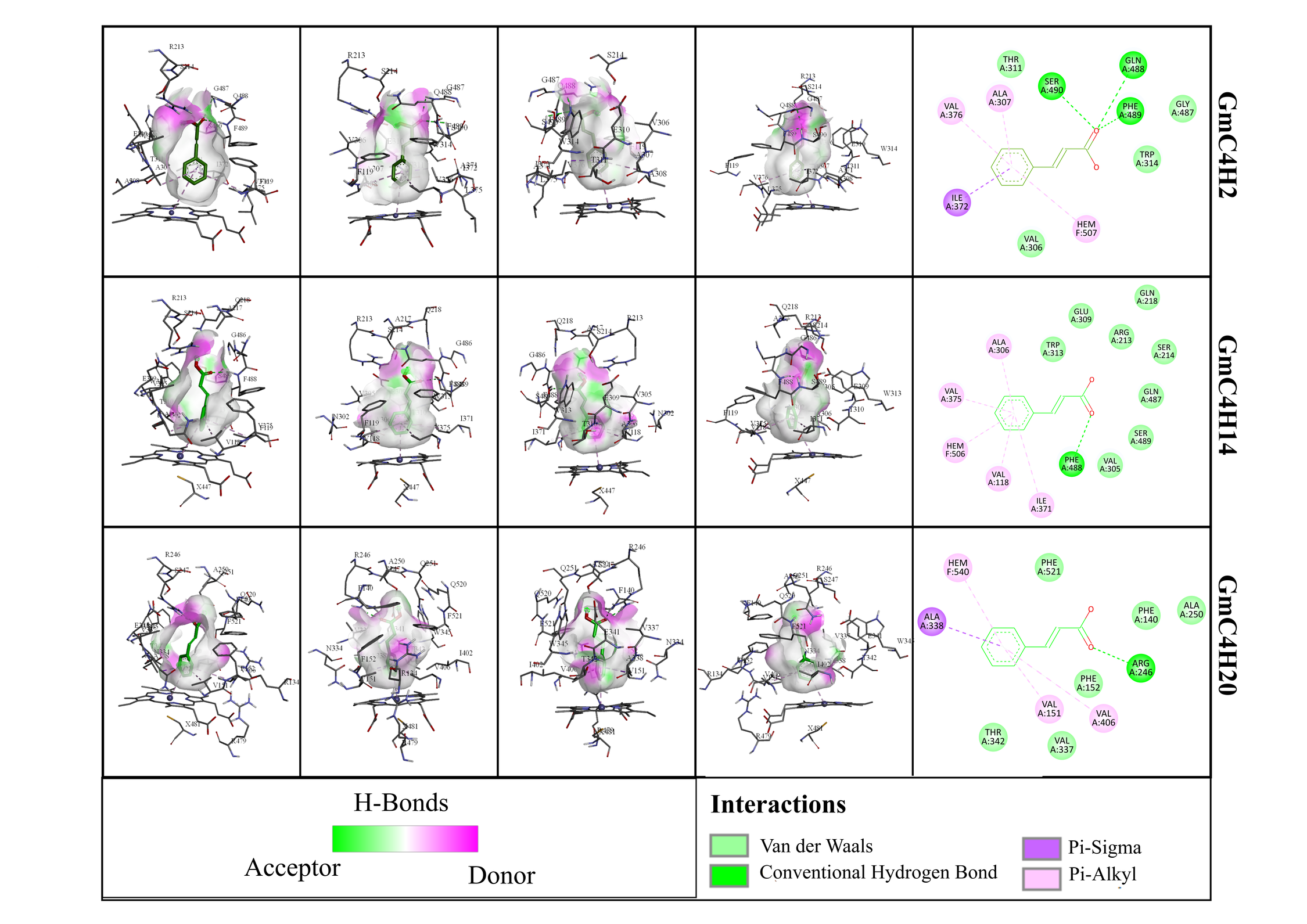

Supplement: S1 Fig — (TIF) [file pone.0285698.s001.tif]
