# Supplementary material for: Functional characterization of Cinnamate 4-hydroxylase gene family in soybean (Glycine max)
Source: PLoS One. 2023 May 15;18(5):e0285698. doi: 10.1371/journal.pone.0285698 (PMC10184913; doi:10.1371/journal.pone.0285698)
Supplement: S1 Table — (DOCX) [file pone.0285698.s002.docx]

**Table S1:** List of Primer used in for vector construction, gene cloning and qPCR

| **Primer name** | **Sequence (5' to 3')** | **Purpose** | |
| --- | --- | --- | --- |
| NotLjCPR1-F | GGGCGGCCGCACTAGTATCGATGGAAGAATCAAGCTCCATGAAG | Vector Construction | |
| PacLjCPR1-R | TTAATTAATCACCATACATCACGCAAATAC |  |  |
| ApaAttR1 | GGGGCCCCATCACAAGTTTGTACAAAAAAGCTGAAC |  |  |
| SacIIAttR2 | GGCCGCGGCACCACTTTGTACAAGAAAGCTGAAC |  |  |
| GmC4H2/14GWF | GGGGACAAGTTTGTACAAAAAAGCAGGCTTCATGGATCTCCTCCTTCTGGAAAAGACCCTC | Cloning of *GmC4H2* and *GmC4H14* | |
| GmC4H2/14GWR | GGGGACCACTTTGTACAAGAAAGCTGGGTC  CTAAAATGACCTTGGCTTTGCCACAATGGTG |  |  |
| Gm20C4H-F-GW | GGGGACAAGTTTGTACAAAAAAGCAGGCTTATGGGTCTTCAAATCAAGGAA | Cloning of *GmC4H20* | |
| Gm20C4H-R-GW | GGG GAC CAC TTT GTA CAA GAA AGC TGG GTC TCATAGTGTCTTAATTGGATGGAACAAC |  |  |
| Gm20C4H-F-GW | GGGGACAAGTTTGTACAAAAAAGCAGGCTTATGGGTCTTCAAATCAAGGAA | Subcellular localization of *GmC4H20* | |
| Gm20C4H-R1-GW | GGGGACCACTTTGTACAAGAAAGCTGGGTcTAGTGTCTTAATTGGATGGAACAAC |  |  |
| GmC4H20F2 | CATCAACGTTGCAGCAATTGAGAC | GmC4H20 | qPCR |
| GmC4H20R2 | TGGTTCACCAACTCTGCTACTGC |  |  |
| GmC4H14F2 | ACACATGAACCTCCACGATGCG | GmC4H14 |  |
| GmC4H14R2 | ATCTTGCTCTCTGCTGGGATGTC |  |  |
| qGmC4H2F | CCAAGAGCACCAACAACAATA | GmC4H2 |  |
| qGmC4H2R | TTCAATTGCAGCAACGTTG |  |  |
