# Supplementary material for: Functional characterization of Cinnamate 4-hydroxylase gene family in soybean (Glycine max)
Source: PLoS One. 2023 May 15;18(5):e0285698. doi: 10.1371/journal.pone.0285698 (PMC10184913; doi:10.1371/journal.pone.0285698)
Supplement: Attachment — Submitted filename: Response to reviewers Comments.docx [file pone.0285698.s004.docx]

**Response to reviewers Comments**

**Reviewer #1:** “Functional characterization of Cinnamate 4-hydroxylase gene family in soybean (Glycine max)” is a massive piece of work and it has provided good information. I thank the authors for this work. The introduction provides comprehensive informations on the background. The research was properly planned and performed. Methods and results are well described. I recommend this manuscript for publication.

**Response:** Thank you

**Reviewer #2:** Khatri et. al characterized the soybean C4H genes and confirmed cinnamate hydroxylation activity by enzymatic assay with heterologously expressed protein. The authors also provided a cloning strategy for the coexpression of a plant CPR with P450 for future functional study. The authors claimed they have identified the C4H genes across the manuscript, which have already been reported previously. They should take less credits on that or just claim the locus ID following reannotated of soybean genome was updated.

**Response:** Thank you for your valuable feedback. The main objective of our study was to characterize the function of C4Hs in soybean and develop a vector that can aid in the characterization of P450s, specifically in legumes. While previous study by Y. Reinprecht et al. (2017) identified putative C4H candidates, our study aimed to provide functional validation of C4Hs in soybean using enzyme assays, as well as to study the dynamics of soybean C4Hs.

Line 275&line 365 The docking was done for GmC4H10 and the authors reported docking energy. Thus, instead of saying “not observed”, it is better to claim the docking position is impossible for hydroxylation.

**Response:** As suggested by the reviewer, we have amened the statement.

Line 308 Specifically, only class II P450 requires CPR.

**Response:** As suggested by the reviewer, we have revised the sentence to emphasize only eukaryotic P450 (class II) require CPR.

Line 322 It could be fairer to compare the catalytic efficiency (Vmax/Km). The full kinetic assay (Michaelis–Menten fitting) with three C4Hs is more informative than figure 7, and figure 7 can be moved to supplementary figure.

**Response:** Thank you for your suggestion regarding the comparison of catalytic efficiency for the three C4Hs in our study. We agree that comparing Vmax/Km is a more comprehensive way of comparing the catalytic activity of the enzymes. Therefore, we have performed the full kinetic assay (Michaelis-Menten fitting) as suggested and the results are consistent with our initial findings. We would like to keep figure 7 in the main paper as it is a visual representation of our finding that one of the three C4Hs is more efficient in soybean, and we believe it adds value to the paper. We hope this will be acceptable.

Line 392 Has the catalytic efficiency been compared to that with soybean CPR?

**Response:** We are unsure of the reviewer’s query here. As per our understanding, it was not possible for us to compare the efficiency with soybean CPRs because we used the product formation measurement method in the enzyme assay instead of the NADPH oxidation method, which can be used to analyze the kinetics of CPRs. In our study, we used *Lotus japonicus* CPR1 for all three C4Hs, and therefore, we believe that the kinetic parameters are comparable. LjCPR1 has been used in several studies as below:

Seki, H., Sawai, S., Ohyama, K., Mizutani, M., Ohnishi, T., Sudo, H., Fukushima, E.O., Akashi, T., Aoki, T., Saito, K., and Muranaka, T. (2011). Triterpene Functional Genomics in Licorice for Identification of CYP72A154 Involved in the Biosynthesis of Glycyrrhizin. *The Plant Cell* 23, 4112-4123.

Yano, R., Takagi, K., Takada, Y., Mukaiyama, K., Tsukamoto, C., Sayama, T., Kaga, A., Anai, T., Sawai, S., Ohyama, K., Saito, K., and Ishimoto, M. (2017). Metabolic switching of astringent and beneficial triterpenoid saponins in soybean is achieved by a loss-of-function mutation in cytochrome P450 72A69. *The Plant Journal* 89, 527-539.

Line 387 There is an underlined o in coumaric acid.

**Response:** Corrected.
